# Larvae and a new species of *Ancyronyx* Erichson, 1847 (Insecta, Coleoptera, Elmidae) from Palawan, Philippines, using DNA sequences for the assignment of the developmental stages

**DOI:** 10.3897/zookeys.136.1914

**Published:** 2011-10-13

**Authors:** Hendrik Freitag, Michael Balke

**Affiliations:** 1 Biology Department, De La Salle University, Taft Avenue 2401, RP-1004 Manila, Philippines and Senckenberg Naturhistorische Sammlungen Dresden, Königsbrücker Landstrasse 159, D-01109 Dresden, Germany; 2 Zoologische Staatssammlung, Münchhausenstrasse 21, D-81247 Munich, Germany and GeoBio-Center, Ludwig-Maximilians-Universität, Munich, Germany

**Keywords:** *Ancyronyx*, larva, *cox1*, DNAbarcoding, new species, taxonomy, AQUA Palawana, Spider Water Beetle, Elmidae

## Abstract

*Ancyronyx montanus* **sp. n.** is described based on adults and larvae, matched using their *cox1* DNA sequence data. Larvae of six additional species of *Ancyronyx* Erichson, 1847 were also described here for the first time, aided by *cox1* or *cob* data: *Ancyronyx helgeschneideri* Freitag & Jäch, 2007, *Ancyronyx minerva* Freitag & Jäch, 2007, *Ancyronyx patrolus* Freitag & Jäch, 2007, *Ancyronyx procerus* Jäch, 1994, *Ancyronyx punkti* Freitag & Jäch, 2007, *Ancyronyx pseudopatrolus* Freitag & Jäch, 2007. *Ancyronyx procerus* is newly recorded from the Philippines by a larval specimen from Busuanga island. The new species and larval stages are described in detail and illustrated by digital and SEM images. A key to the *Ancyronyx* larvae of Palawan and an updated checklist of Philippine *Ancyronyx* is provided.

## Introduction

The genus *Ancyronyx*, usually referred to as spider water beetle, belongs to the predominantly aquatic riffle beetle family Elmidae Curtis, 1830 (Coleoptera), subfamily Elminae Curtis, 1830. Ancyronychini Ganglbauer, 1904 has been erected exclusively for this genus which is known from North America and Southeast Asia. The adults have extremely long legs and strong claws as an adaptation to their riverine habitats. Elmidae are often highly sensitive to water pollution and are therefore of great value as bioindicators (e.g. Moog and Jäch 2003; Hilsenhoff 1982). This requires, however, taxonomic knowledge and appropriate identification tools. *Ancyronyx* larvae were unknown until Brown (1972) illustrated those of the North-American species *Ancyronyx variegatus* (Germar, 1824). Philippis (1997) published the first data on the life cycle and growth of this species. He successfully used head capsule width to assign the respective instar stage to the larvae.

Publications dealing with molecular data of Elmidae are very rare and DNA data are only available for one *Ancyronyx* species, *Ancyronyx procerus* Jäch, 1994 (Čiampor & Ribera 2006).

The first *Ancyronyx* speciesfrom the Philippines was recorded and described by Jäch (1994). Subsequently, seven new species were added (Jäch 2004, Freitag and Jäch 2007) and more new species await description. Currently, the Philippine province of Palawan is very well sampled with regard to Elmidae due to the AQUA Palawana Program (http://aquapalawana.nhm-wien.ac.at) conducted by the first author for more than 10 years. The copious collection of *Ancyronyx* specimens, both adult and larvae, retrieved by this taxonomic research initiative gave us the opportunity to study the larvae of this genus taxonomically for the first time.

## Material and methods

### Taxon sampling

The larvae examined were partly collected by standardized methods for ecological studies such as colonization and drift sampling during a survey in 2000 / 2001 (Freitag 2005). Such samples are referred to by the letters “C” (colonization sample) or “D” (drift sample), while manual samples are indicated by “M” at the end of a collection label. This variety of methods appeared to be more successful to retrieve rare species (Freitag, 2008). Most larval material of *Ancyronyx helgeschneideri* was obtained this way. However, the collected samples were preserved in formalin, thus the materials are consequently not suitable for molecular-genetic analysis.

**Figure 1.**
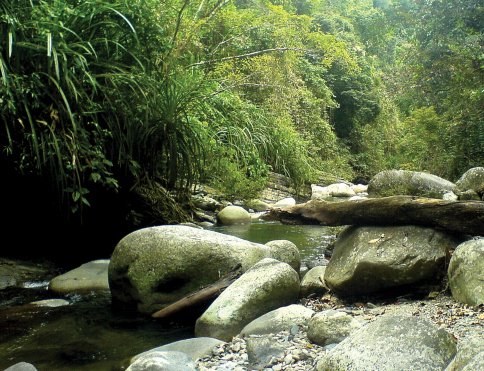
Small to medium sized, permanent forest streams with heterogeneous morphology and rich in submerged woods and root packs are usually inhabited by the highest numbers of *Ancyronyx* species. This picture shows the type locality of *Ancyronyx montanus* where also *Ancyronyx minerva*, *Ancyronyx patrolus*, *Ancyronyx pseudopatrolus* and *Ancyronyx punkti* occurred sympatrically.

All materials from more recent surveys have been retrieved by means of manual collection from submerged wood debris or through the use of a fine-meshed hand net. This material was preserved in absolute ethyl alcohol and thus, it was suitable for genetic sequencing. The best manual sampling was possible in permanent small to medium sized rivers in forested area (Fig. 1).

The label codes for the sampling sites of the first author are arbitrary. They do not follow any temporal or spatial pattern, except for the fact, that eventually varying small letters following a common code number refer to different sections of the same water system.

### DNA extraction and sequencing

**Figure 2.**
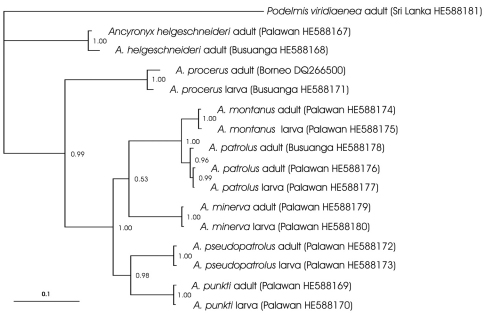
Phylogram of the consensus tree of the Bayesian analysis with branch lengths measured in expected substitutions per site. Posterior probability values (printed when > 0.5) at respective branches. Sample labels with island of origin and code of the collection site or genbank code.

DNA was extracted from 15 whole specimens (Fig. 2) using Qiagen DNeasy kit (Qiagen, Hilden, Germany) and a single elution following the protocol for animal tissues (Qiagen 2002). The 3’ end of the cytochrome *c* oxidase subunit I (*cox1*) gene was amplified using polymerase chain reaction (PCR) following standard protocols (e.g. Caterino et al. 2005) and using primer pairs C1-J-2183 (5’-caa cat tta ttt tga ttt ttt gg-3’; *Jerry*) and TL2-N-3014 (5’-tcc aat gcs cts atc tgc cat aat a-3’; *Pat*) (Simon et al. 1994) and *Mango Taq* DNA polymerase (Bioline, Luckenwalde, Germany). The PCR temperature progression was: 30 s at 94°C, 30 s at 47°C, 60 s at 72°C (× 35 cycles), 600 s at 72°C. Amplification products were purified with Qiagen Qiaquick PCR purification columns (Qiagen, Hilden, Germany). Cycle sequencing was performed as follows: 15 s at 96°C, 15 s at 50°C, and 240 s at 60°C (× 35 cycles) using PCR primers with BigDye Terminator v3.1 Cycle Sequencing Kit (Applied Biosystems, Foster City, California, USA). The sequencing products were purified by ethanol precipitation (25 µl of cold (-20°C) 99% ethanol, 2.5 µl of 3M sodium acetate added to product; centrifuged; washed with 25 µl of 70% ethanol), and additionally with Agencourt CleanSEQ (Agencourt Bioscience, Beverly, Massachusetts, USA) following protocol 000600v32 (Agencourt Bioscience 2006) before electrophoresis.

Additionally, a central part of the cytochrome b apoenzyme (*cob*) gene of four specimens was amplified as described above by using the primer pair 5’-gag gag caa ctg taa tta cta a-3’ (CB3) and 5’-aaa aga aa(ag) tat cat tca ggt tga at-3’ (CB4) (Baraclough et al. 1999). This was done to support the matching of a larva for which *cox1* data were not available.

### Phylogenetic analysis

*Podelmis viridiaenea* Jäch, 1982 (Elmidae: Elminae: Elmini) from Sri Lanka was used as an outgroup. Two additional sequences of *Ancyronyx procerus* were retrieved from GenBank: DQ266500.1, DQ266511.1 (Čiampor and Ribera 2006). Sequences were edited and aligned in CLUSTALW (Thompson et al. 1994) using BIOEDIT version 7.0.5.2. (Hall 1999) and default parameters. Phylogenetic analyses were conducted with MRBAYES vers. 3.1.2 (Ronquist and Huelsenbeck 2003) using the GTR (General Time Reversible) model (Tavaré 1986) with default priors starting with random trees with three heated and one cold Markov chains. The analysis was run by 1,000,000 generations, 7,501 trees were sampled after the first 25% of samples from the cold chain have been discarded as burnin. Branch support for the Bayesian trees was assessed with posterior probabilities determined via the 50% majority rule consensus. This “quick” analysis was sufficient for the task of detecting which larva might be associated with which adult.

### Morphological analysis

Scanning electron microscope (SEM) images were obtained using a ZEISS EVO 50 XVP at SMTD. The specimens were coated with gold using two samples each if enough material was available. Those taxa of which only a single or few specimens have been available were only vacuum dried, but not gold-coated prior to scanning. This resulted in lower quality of the micrographs, but has kept the specimen´s surface natural.

Digital habitus photographs were taken with a NIKON SMZ800 stereo microscope with digital photo adapter NIKON DS-Fi1 (unit in DLSU). These photographs were taken at various focus layers and were subsequently combined using COMBINEZM software (Hadley 2008) to retrieve images with sufficient depth of focus. The same system was used for the dissection of adult specimens and the detailed material examination.

Examination, biometric measuring and imaging of dissected parts were conducted using a NIKON Eclipse 600 microscope with a ZEISS AxioCam MRc5 digitalcamera (unit in SMTD). The morphological details of larvae are described from external view as they are usually visible without dissection, if not stated opposite. The listing of examined material includes the head width (in mm) of all measured larvae.

Morphological terminology used herein mainly follows the Elmidae chapter of the recently published Handbook of Zoology / Coleoptera (Kodada and Jäch 2005).

### Abbreviations

asl	above sea level (altitude)

C	colonization sample

CL 	calculated length (PL + EL)

CPOM	Coarse Particulate Organic Matter

D	drift sample

EL 	elytral length

EW 	elytral width

ex. / exs.	exemplar / exemplars of adult specimen

FPOM	Fine Particulate Organic Matter

HW 	head width

ID 	interocular distance

L	larva / larvae

M 	manual collection

MW 	maximum pronotal width

PL 	pronotal length

CFP	H. Freitag private collection, Dresden, Germany

IMRL 	Phyllodrom, Institut und Museum für Regenwaldökologie Leipzig, Germany

NMW 	Natural History Museum Vienna, Austria

MNCN	Museo Nacional de Ciencias Naturales Madrid, Spain

PCSD	Palawan Council for Sustainable Development, Philippines

SMTD 	Senckenberg Museum für Tierkunde Dresden, Germany

UPLB 	University of the Philippines Los Baños, Museum of Natural History, Entomological Collection, Philippines

ZMUC	Zoological Museum of the University Copenhagen, Denmark

ZSM 	Zoological State Collections Munich, Germany

## Results

### DNA sequence analysis

Alignment of the *cox1* data and trimming ambiguous bases at the 3’ and 5’ ends yielded a matrix of 770 bp. None of the sequences contained indels. The sequence of the larvae of *Ancyronyx pseudopatrolus* had ambiguous six positions in-between, that were coded as ‘N’s.

The sequence divergence between *Ancyronyx* species was between 3.3.% (25 positions different: *Ancyronyx patrolus* / *Ancyronyx montanus* ) and 18% (139 positions different: *Ancyronyx helgeschneideri* / *Ancyronyx montanus*). The highest divergence with another taxon (17%–20%) was with the outgroup species in all cases.

All adults and larvae could be matched unambiguously. Sequences of adult and larva of the same species from the same locality or island showed low difference of positions in most species (2–3 positions different, 0.2–0.4% divergence). *Ancyronyx pseudopatrolus* would be within this range when assumed that all unidentified positions are not varying between adult and larva, which is likely as these positions appear to be conserved sites in *Ancyronyx*. Sequence samples of the same species, but from different islands showed slightly higher divergence of 0.6%–2.1% (5–16 positions). A 50% majority rule consensus trees based on *cox1* data is illustrated in Fig. 2.

The sequencing of *cox1* of the one and only available alcohol-preserved larva of *Ancyronyx helgeschneideri* failed and is consequently not included in the phylogenetic analyses. However, we were able to amplify the *cob* sequence of that larval specimen and match it with its adult stage. Their aligned *cob* sequences of 378 bp were identical except for two positions of synonymous exchange of a base (0.5% divergence). In *Ancyronyx procerus* six positions of the *cob* sequences varied between adult and larval specimens by synonymous changes (1.6% divergence). Both species varied in 61 positions of their *cob* sequence (16% divergence).

All sequences were submitted to GENBANK. Accession numbers and curatory information are listed in Table 1.

**Table1.** Genbank accession numbers of DNA sequences, geographical origins, collectors, collection sites and organismic sample references of specimens used for molecular-genetic analyses.

Species	Stage	Locality	Site	Collector	Voucher	*cox1*	*cob*
*Ancyronyx montanus* Freitag & Balke, sp. n.	adult	Palawan	16h	Freitag	NMW FR 037	HE588174	-
*Ancyronyx montanus* Freitag & Balke, sp. n.	larva	Palawan	16h	Freitag	ZSM FR 038	HE588175	-
*Ancyronyx minerva* Freitag & Jäch, 2007	adult	Palawan	154	Freitag	ZSM FR 001	HE588179	-
*Ancyronyx minerva* Freitag & Jäch, 2007	larva	Palawan	159	Freitag	ZSM FR 025	HE588180	-
*Ancyronyx punkti* Freitag & Jäch, 2007	adult	Palawan	154	Freitag	ZSM FR 008	HE588169	-
*Ancyronyx punkti* Freitag & Jäch, 2007	larva	Palawan	154	Freitag	ZSM FR 002	HE588170	-
*Ancyronyx pseudopatrolus* Freitag & Jäch, 2007	adult	Palawan	16f	Freitag	ZSM FR 003	HE588172	-
*Ancyronyx pseudopatrolus* Freitag & Jäch, 2007	larva	Palawan	16b	Freitag	ZSM FR 040	HE588173	-
*Ancyronyx patrolus* Freitag & Jäch, 2007	adult	Busuanga	165	Freitag	ZSM FR 032	HE588178	-
*Ancyronyx patrolus* Freitag & Jäch, 2007	adult	Palawan	20	Freitag	ZSM FR 005	HE588176	-
*Ancyronyx patrolus* Freitag & Jäch, 2007	larva	Palawan	20	Freitag	ZSM FR 006	HE588177	-
*Ancyronyx helgeschneideri* Freitag & Jäch, 2007	adult	Busuanga	169	Freitag	ZSM FR 013	HE588168	-
*Ancyronyx helgeschneideri* Freitag & Jäch, 2007	adult	Palawan	CR4	Freitag	ZSM FR 007	HE588167	HE588183
*Ancyronyx helgeschneideri* Freitag & Jäch, 2007	larva	Palawan	CR4	Freitag	ZSM FR 061	-	HE588184
*Ancyronyx procerus* Jäch, 1994	adult	Borneo		Čiampor	MNCN FC-B05	DQ266500	DQ266511
*Ancyronyx procerus* Jäch, 1994	larva	Busuanga	169	Freitag	ZSM FR 014	HE588171	HE588182
*Podelmis viridiaenea* Jäch, 1982	adult	Sri Lanka	1	Freitag	ZSM FR 035	HE588181	-

## Taxonomy

### Ancyronyx minerva

Freitag & Jäch, 2007

http://species-id.net/wiki/Ancyronyx_minerva

Figs 3 11A–L

Ancyronyx minerva  Freitag & Jäch, 2007: 50–53 (adult description); Freitag and Pangantihon 2010: 133 (first record Mindoro).

#### Material examined. 

2L (0.21, 0.24) (PCSD) “PHIL.: Palawan, P. Princesa Panaguman R., Marufinas 10°15'09"N, 118°58'03"E 15.6.2001, leg. Freitag (PR1)C-R".1♀ (CFP) “PHIL.: Palawan, P.Princesa; Concepcion, Tagpaya, Camp Aga, Taranaban R. trib.; mount.creek c.8km upstr. Highw., dist. prim. forest; riffle; rocks, boulders, roots; c.450m asl 10°05'N, 119°01'E, 26.4.1995 leg. Pangantihon (16f)M“; 2L (0.28, 0.29) (CFP) “PHIL.:Palawan, P.Princesa, Sta. Cruz, Calatobong Riv., Nat.Highw. km29; rocks CPOM, riffe & side pools; heavy metal soil,sec.veget.,25m asl, 9°56'41"N, 118°44'56"E 14.11.1995, leg.Freitag (124)"; 42 exs, 3L (0.27, 0.28, 0.30) (PCSD, NMW) “PHIL.: Palawan, P. Princesa, Iwahig, Balsahan Riv., upstr.dam; riffle, boulders, gravel, wood, moss; 9°46'36"N, 118°39'55"E 24.1.1995, leg. Freitag (20)M"; 2L (2 × 0.27) (CFP) “PHIL.: Palawan, P. Princesa, Irawan River, 6km NW of PPC, 2km upstream of water plant 9°49'N, 118°39'E 06.4.1994, leg. Freitag (60)M"; 1L (0.27) (ZSM [FR025]) “PHIL.: Palawan, P. Princesa, Iwahig, Salomon Riv.; rural near sec. forest, riffle, boulders, gravel, wood; 9°46'59"N, 118°40'53"E 24.1.1994, leg. Freitag (159)M"; 2♂♂, 1♀, 4exs., 1 L (0.26) (CFP) “PHIL: Palawan, P.Princesa, Bgy. Montible, rd. km 29, Iwahig River, big mount. river, boulders, CPOM; 9°41'20"N, 118°37'29"E 24.11.2007, leg. Freitag (130)M“; 1♀, 4 exs., 1 L (0.24) (ZSM [FR001], ZMUC) “PHIL.: Palawan, Aborlan; Cabigaan, Talakaigan R.; mount.Riv. upst.dam, riffle, rocks, boulders, CPOM, forest, c.50m asl, 9°26'50"N, 118°26'49"E 25.2.1995, leg. Freitag (154)M“; 2♂♂, 1♀, 11 L (2 × 0.22, 2 × 0.25, 2 × 0.26, 3 × 0.27, 2 × 0.28) (SMTD) “PHIL.: Palawan, Narra, 7 km N town centre, downstr. Estrella Falls, mountain riv.; sec. forest; gravel, boulders, submerged wood, riffle; c. 50m asl., c. 9°20'N, 118°23'E 16.4.2010, leg. Freitag & Pangantihon (180a)“; 12 exs. (CFP) “PHIL.: Palawan, Narra, 5 km W town proper, Taritien River, riffle, boulders, leaf litter; c. 100m asl. 9°19'11"N, 118°22'35"E 17.4.2010, leg. Freitag et al. (182a)“; 1♂ (CFP) “PHIL.: Palawan, Rizal, Campung-ulay, Kalitawan Riv.; HW km 212.2, sec. veget/forest; slightly polluted, submerged wood in run, c. 30m asl. 9°19'11"N, 118°22'35"E 02.7.2010, leg. Freitag (186)"; 7 exs. (ZSM) “PHIL.: Palawan, Rizal, Ransang, Ransang Riv., E of HW km 223; sec. veget. Caingin; submerged wood, c. 50m asl. 9°19'11"N, 118°22'35"E 02.7.2010, leg. Freitag (184b)“; 1♀ (CFP) “PHIL.: Palawan, Rizal, Punta Baja, Malambunga Riv.; HW km 202.1, farmland; submerged wood in run, c. 10m asl. 9°19'11"N, 118°22'35"E 02.7.2010, leg. Freitag (187a)”;

**Figure 3–10.**
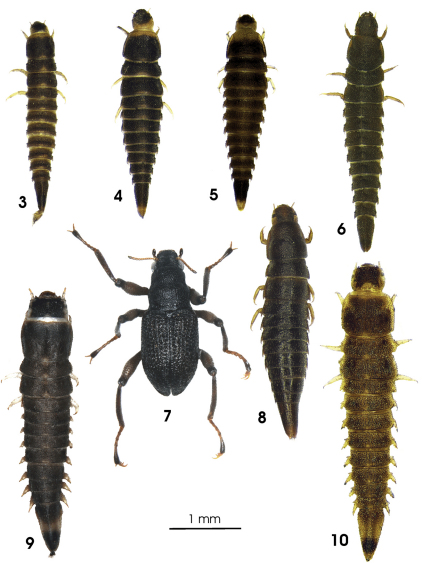
Habitus of **3** *Ancyronyx minerva* Freitag & Jäch, 2007, larva, **4** *Ancyronyx punkti* Freitag & Jäch, 2007, larva, **5** *Ancyronyx pseudopatrolus* Freitag & Jäch, 2007, larva, **6** *Ancyronyx patrolus* Freitag & Jäch, 2007, larva, **7** *Ancyronyx montanus* Freitag & Balke, sp. n., adult, **8** *Ancyronyx montanus* Freitag & Balke, sp. n., larva, **9** *Ancyronyx procerus* Jäch, 1994, larva, **10** *Ancyronyx helgeschneideri* Freitag & Jäch, 2007, larva.

#### Larval description (based on 6^th^ instar). 

Colour as in Fig. 3, predominantly dark-brown; legs, antennae, posterior and lateral head portions yellowish. Most anterior portion of pronotum with yellowish band reaching up to the lateral margins, this pale band slightly extended posteriad along the midline. Meso- and metanotum and abdominal segments at medioposterior margin with broadly subtriangular yellow pattern. A light colour pattern might by present or lacking at the apex of abdominal segment IX, but never extends anteriad up to more than 0.2 posterior .

**Figure 11.**
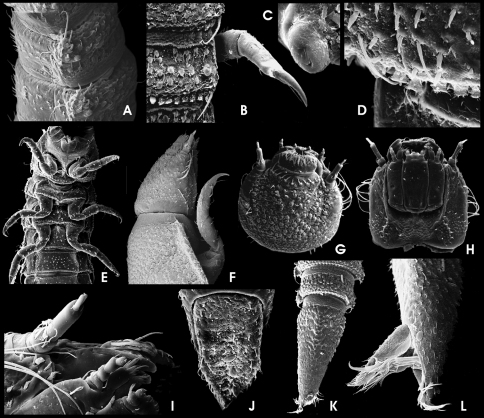
*Ancyronyx minerva* Freitag & Jäch, 2007, larva (SEM photographs), **A** abdominal segments, lateral, with posterolateral projections and spiracles, **B** posterior portion of metanotum and first abdominal segment, dorsal, with different setae, posterolateral trichoid tooth and spiracle, **C** abdominal spiracle, **D** posterolateral detail of abdominal segment V, dorsal, with different types of setae, **E** thoracic and first abdominal segments, ventra, **F** head and pronotum, lateral, **G** head, dorsal, **H** head, ventral, **I** antenna and distal parts of maxilla, labium and mandible, ventral, **J** operculum, ventral, **K** abdominal segments VIII and IX, dorsal, **L** distal portion of abdominal segment IX, lateral, with opened operculum, abdominal gill chamber and terminal hooks.

HW c. 0.27 mm; entire larva about 3.0 mm long. Body torpedo-like elongate, subsemicircular in cross section, dorsally vaulted, ventrally almost flat. Posterolateral margins of abdominal segments I–VIII moderately produced (Fig. 11A). These projections either not, or only slightly overreaching posterior segment margins. Posterolateral edge of these projections with long posteriad-directed trichoid tooth, overreaching middle of subsequent abdominal segment when that retracted (11B). Spiracles (Figs 11A–C) present laterally on mesothorax and abdominal segments I–VIII, surrounded by glabrous areas. Dorsal side densely covered with setiferous tubercles (Fig. 11B). Ventral side smoother, with scattered setae (Fig. 11D). Retractable portions of body segments without setae and tubercles (Figs 11A, E).

Head (Figs 3; 11F–I) exposed, distinctly prognathous, sides subparallel in posterior half, with lateral clumped, not exposed stemmata in a glabrous area, lined by a irregular row of six long acuminate setae on each side (Fig. 11F). Frontal suture U-shaped, rather inconspicuous; frontoclypeal suture arcuate (Fig. 11G). Clypeus distally glabrous, with subbasal fringe of fasciculate setae originated from tubercles (Fig. 11G). Ventral side (Fig. 11H) with almost glabrous median portion, lateroposteriorly with tubercles. Gula with concentrically arranged asperities. Antenna (Fig. 11I) three-segmented, c. 1/3 as long as head, basally connected to a segment-like membranous peduncle. Peduncle stout, bald, partly retracted into head capsule; scape slightly longer than peduncle about as long as broad, with dorsolateral fringe of branched trichoid setae; pedicel cylindrical, more than two times as long as scape and c. three times as long as broad, with few apical trichoid setae; flagellum and sensorium cylindrically elongate, subequal in length; apex of flagellum with cylindrically elongate sensillum. Labrum (Fig. 11G) subrectangular, c. 2.5 times as wide as long, with a subapical fringe of ramose setae and scattered trichoid and truncate (sensory) setae, proximal portion glabrous. Mandibles with distal portion subfalcate; apex tridentate; incisory margin densely setose; outer margin glabrous, with one elongate, ramose squamose seta (Fig. 11I). Maxilla (Figs 11H, I) moderately broad; cardo stout, divided, lateral portion with one median lanceolate seta; stipes subtrapezoidal, glabrous, with few lanceolate and one latero-subapical trichoid seta; maxillary palpus four-segmented, approx. as long as stipes broad, distal segment subglobular with several apical sensilla of various shape; predistal segment with lateroapical trichoid seta; galea and lacinia subequal in length and shape, shorter than palpus, apically with acuminate setae. Labium as in Figs 11H, I; mentum (postmentum) broad (about 1.5 times of stipes), with median groove most depressed posteriorly, with one pair of moderately long trichoid setae sublaterally at anterior 0.25, one pair of subbasal spinose setae and one pair of short subapical lateral spines; submentum (prementum) short, subrectangular, with one laterobasal pair of setae; ligula inconspicuous with various setae and pegs; labial palpi short, with short and stout palpifer; apical segment similar to that of maxillary palpi, preapical segment with lateral tuft.

Pro-, meso- and metathorax (Figs 3; 11E, F) subtrapezoidal, slightly narrower anteriorly, broader than long; with lateral rim distinctly produced laterad. Pronotum longest, with rather inconspicuous round signa (glabrous areas) in posterior half. Meso- and metathorax distinctly shorter than prothorax. Venter of prothorax (Fig. 11E) with five sclerites: two oblique anterior (fused episternum and basisternum), two lateral (pleuron), and one posteromedial sclerite (sternellum), posterior portions of lateral sclerites strongly extended mesad, meeting posteromedial sclerite; posteromedial sclerite with posterior fringe of short setae; procoxal cavity closed posteriorly; anterior and lateral portions glabrous, few setiferous tubercles in posterior portions. Venter of meso- and metathorax (Fig. 11E) with six sclerites: two large anterior (divided basisternum), two subcircular sclerites anterolateral (divided pleuron), two meso-posterolateral sclerites (divided pleuron); coxal cavities open posteriad; setiferous tubercles sparse on median portion; lateral portions almost glabrous. Posterior margin of anterior sclerites with fringe of setiferous tubercles.

Legs (Figs 11E, F) moderately long (compared to larvae of other genera), but much shorter than in adults, similar in shape and length, with scattered ramose squamose sensilla and additional trichoid sensilla at femora and tibiae. Coxae large, subtrapezoidal; trochanter shorter, subtriangular; femora subconical, broadest distal; tibiae subcylindrical, longest segment, narrower than femur, broadest basally. Claws elongate, strongly bent (basal to distal part in rectangular angle) with one subbasal trichoid tooth.

Abdomen (Figs 3; 11A–D, J–L). Segment IX with large, ventral, subtrapezoidal operculum (Fig. 11J). Segments I–VIII similar in shape, subrectangular in dorsal view. Retractable anterior portion with squamose asperities (Fig. 11A); posterior margin dorsally and ventrally with a rim of squamose setae (Fig. 11D). Remaining median portions of terga covered with setiferous tubercles, most densely at the sagittal area, forming slightly elevated dorsosagittal carinae at the posterior portions of segments I–VIII and the subanterior portion of segment IX (Fig. 11K). Ventral sclerites of segments I–VIII subrectangular, increasingly fused with pleural sclerites from 1^st^ to 8^th^ segment (pleural sclerites not distinguishable in segments VII and VIII); ventral sclerite of segment I with sagittal ridge in anterior third (Fig. 11E). Segment IX (Figs 11J–L) elongate, conical, apex not emarginate. Operculum (Fig. 11J) c. double as long as broad, medially depressed, rugose, laterally with rim of trichoid setae, with a pair of hooks inserted at median dorsal (inner) side (Fig. 11L). Gill chamber with long, ramose gill tufts overreaching the opercular margin (Fig. 11L).

#### Variation between larval instars. 

Almost the entire material studied belongs to the final instar stage. The two presumably prefinal instar specimens do not vary conspicuously from the description above. The legs appear slightly broader and shorter in relation to the body and their setae patterns are slightly different.

#### Distribution.

Only known from Palawan and Mindoro (Freitag & Pangantihon, 2010).

### Ancyronyx punkti

Freitag & Jäch, 2007

http://species-id.net/wiki/Ancyronyx_punkti

Figs 4 12A–K

Ancyronyx punkti  Freitag & Jäch, 2007: 47–50 (adult description).

#### Material examined. 

1♀ (ZMUC) “PHIL.: Palawan, P.Princesa; Bgy. Binduyan, Olanguan Falls/Brdg., Nat. Highway km 29; 2.5 km upstr.,rocks, gravel boulder, CPOM; degr. prim. veget.,10°02'11"N, 119°03'01"E 28.08.2010, leg. Freitag (126a)M"; 3 exs. (CFP) PHIL.: Palawan, P.Princesa; 2km SE Laptay/Napsan, Bubugtungan mountain stream, semi-prim.forest, 9°41'14"N, 118°27'17"E 07.9.2008, leg. Freitag (23a)M; 2 exs., 1L (0.27) (CFP) “PHIL.: Palawan, P.Princesa; Concepcion, Taranaban R.; c.6km upstr. Highw., mount.riv., riffle; boulders, woodlitter; c.150m asl 10°02'30"N, 119°00'45"E, 20.1.1995 leg. Freitag (16b)M"; 5♂♂, 6♀♀, 2 L (0.28, 0.31) (ZSM [FR002, FR008], CFP) “PHIL.: Palawan, Aborlan; Cabigaan, Talakaigan R.; mount.Riv. upst.dam, riffle, rocks, boulders,CPOM,forest, c.50m asl, 9°26'50"N, 118°26'49"E 25.2.1995, leg. Freitag (154)M"; 34 exs., 3L (0.29, 0.30, 0.31) (NMW, SMTD) “PHIL.: Palawan, Narra, 7 km N town centre, downstr. Estrella Falls, mountain riv.; sec. forest, gravel, boulders, submerged wood, riffle; c. 50m asl., c. 9°20'N, 118°23'E 16.4.2010, leg. Freitag & Pangantihon (180a)"; 1♂, 2L (0.20, 0.28) (CFP) “PHIL.: Palawan, Narra, 5 km W town proper, Taritien River near DENR Station; riffle, boulders, gravel, wood litter 9°18'55"N, 118°22'56"E 17.4.2010, leg. Freitag et al. (182b)M"; 37exs., 13L (0.24, 0.25, 0.26, 2 × 0.28, 4 × 0.30, 2 × 0.31, 2 × 0.32) (NMW, ZMUC, IMRL) “PHIL.: Palawan, Narra, 5 km W town proper, Taritien River, riffle, boulders, gravel, wood litter/leaf litter c. 100m asl. 9°19'11"N, 118°22'35"E 17.4.2010, leg. Freitag et al. (182a)"; 3 exs. (CFP) “PHIL.: Palawan, Rizal, Campung-ulay, Kalitawan Riv.; HW km 212.2, sec. veget/forest; slightly polluted, submerged wood in run, c. 30m asl. 9°19'11"N, 118°22'35"E 02.7.2010, leg. Freitag (186)"; 2L (0.29, 0.32) (CFP) “PHIL.: Palawan, Brookes P., Salogon, Manguguran Riv./Sitio, mount. riv.; rocks, CPOM, sand; riffle sec. veget., 70m asl., 8°44'07"N, 117°42'15"E 01.9.1994, leg. Freitag (148)M"; 1 L (0.31) (PCSD) “PHIL.: Palawan, Brookes P., Sabsaban River, c.10km NW of town proper, downstr. falls, grassbank rills, 8°49'N, 117°48'E 20.5.1994, leg. Freitag (79c)M".

#### Larval diagnosis (based on 6^th^ instar).

Colour (Fig. 4) very similar to that of *Ancyronyx minerva*, but most distinctly different by ventral head portions pale; anterior yellow pronotal band not extended posteriad along the midline; meso-, metanotum and abdominal segments with rather inconspicuous or without yellow pattern at medioposterior margin and apex of abdominal segment IX with conspicuous yellow pattern extending anteriad up to c. posterior 0.3.

**Figure 12.**
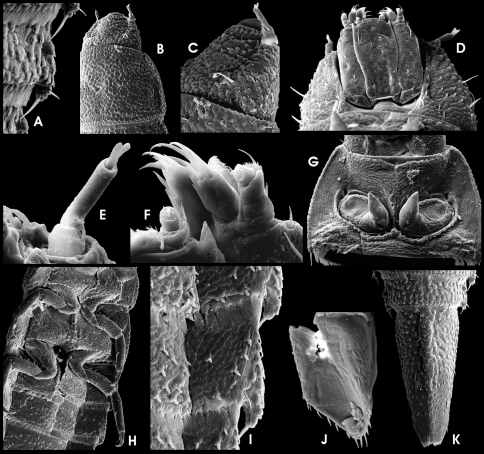
*Ancyronyx punkti* Freitag & Jäch, 2007, (SEM photographs) larva: **A** Posterolateral detail of abdominal segment VI, dorsal, with with posterolateral projections and spiracles, **B** pronotum and head, dorsal, **C** head, dorsolateral, **D** head, ventral, **E** antenna, ventral, **F** apical portions of labium and maxilla, ventral, **G** prothorax, ventral, **H** thoracic and first abdominal segments, ventral, **I** lateral and parts of median sclerites of abdominal venter, with asperities, setiferous tubercles and lateral projections, **J** operculum, lateroventral, **K** abdominal segments VIII and IX, dorsal.

HW 0.32 mm; entire larva about 3.0 mm long. Body elongate, broader than that of *Ancyronyx minerva*, but very similar in the external characters, except for the following:

Posterolateral projections (Fig. 12A) of abdominal segments IV–VIII distinctly overreaching posterior segment margins (approximately as long as the squamose setae at posterior segment margins).

Head (Figs 4; 12B–F) broadest posterior 0.4, not subparallel in posterior half; lateral setae moderately long; dorsolaterally with a pair of rather long double setae (Fig. 12C). Frontal suture V-shaped, rather inconspicuous (Fig. 4). Subbasal fringe of clypeus with rather short fasciculate setae. Ventral side (Fig. 12D) dominantly rugulose, without glabrous areas. Antennae as in Fig. 12E, fringe of scapus setae fasciculate with one long trichoid medial extension each, pedicel longer than in *Ancyronyx minerva* (c. four times as long as broad); apex of flagellum with cylindrically elongate sensillum (broken off in figured specimen). Maxilla (Figs 12D, F) with stipes slightly tapering towards apex; maxillary palpus (Fig. 12F) slightly broader and stouter than in *Ancyronyx minerva*. Labial mentum (Figs 12D, F) distinctly concave in posterior half, narrowest at basal 0.25, pair of trichoid setae long (reaching anterior margin) inserted sublaterally at anterior 0.2; lateroapical pair of spines broad, subtriangular, positioned at distal edge, not subapical. Submentum (prementum), ligula and labial palpi as in *Ancyronyx minerva*.

Pro-, meso- and metathorax (Figs 4; 12G, H) distinctly narrower anteriorly. Pronotum with several rather inconspicuous small round signa (glabrous areas) in posterior half (Fig. 12B). Sclerites of venter of prothorax (Fig. 12G) with concentrically arranged asperities and therefore rugulose, not glabrous. Venter of meso- and metathorax (Fig. 12H) rugulose due to dense cover with asperities.

Legs (Fig. 12H) slightly slenderer than those of *Ancyronyx minerva* and with asperities at coxae, trochanter and femora.

Abdomen (Figs 4; 12A, H–K) without noticeable dorsosagittal carinae except for subanterior portion of segment IX (Fig. 12J); squamose setae at posterior rim of segments I–VIII large and overlapping (Fig. 12I). Ventral sclerites of segment I with sagittal ridge almost reaching up to posterior margin, distinctly longer than 1/3 of segment length (Fig. 12H)

Operculum (Fig. 12J) with deep median depression. Apex of segment IX (Fig. 12K) truncate to very slightly emarginate.

#### Variation between larval instars. 

The few available prefinal instar specimens vary from the description above by relatively slenderer thoracic and abdominal segments, the relatively longer posterolateral projections, the distinctly shorter and broader legs and fewer setae on tibiae and femora.

#### Larval differential diagnosis. 

The species can most easily be distinguished from *Ancyronyx minerva* which looks superficially most similar by the colour pattern of ventral head, pronotum and last abdominal segment and the longer median crest of the first abdominal segment venter.

#### Distribution. 

Known from Palawan island.

### Ancyronyx pseudopatrolus

Freitag & Jäch, 2007

http://species-id.net/wiki/Ancyronyx_pseudopatrolus

Figs 5 13A–E 14A–I

Ancyronyx pseudopatrolus  Freitag & Jäch, 2007: 46–47 (adult description).

#### Material examined. 

11L (2 × 0.17, 2 × 0.22, 0.23, 0.24, 3 × 0.27, 0.28, 2 × 0.29) (NMW, ZMUC, CFP) “PHIL.: Palawan, P. Princesa Panaguman R. 10°15'09"N, 118°58'03"E 17.5.2001, leg. Freitag (PR1)D"; 2L (0.23, 0.27) (PCSD) “PHIL.: Palawan, P. Princesa Panaguman R., Marofinas 10°15'09"N, 118°58'03"E 01.8.2001,leg.Freitag(PR1)C-R"; 2L (0.24, 0.28) (SMTD) “PHIL.: Palawan, P. Princesa Panaguman R., Marofinas 10°15'09"N, 118°58'03"E 15.6.2001, leg.Freitag(PR1)C-R"; 3♂♂, 1♀ (UPLB, ZSM [FR003], PCSD, IMRL) “PHIL.: Palawan, P.Princesa; Concepcion, Taranaban R. trib.; mount.creek c.8km upstr.; dist. prim. forest; riffle;rocks, boulders,roots; c.450m asl 10°05'N, 119°01'E, 28.1.1995 leg. Freitag (16f)M"; 3♂♂, 2♀♀ 1L (0.29) (SMTD, ZSM [FR040], NMW, ZMUC, CFP) “PHIL.: Palawan, P.Princesa; Concepcion, Taranaban R.; c.6km upstr. Highw., mount.riv., riffle; boulders, woodlitter; c.150m asl 10°02'30"N, 119°00'45"E, 20.1.1995 leg. Freitag (16b)M".

#### Remarks. 

**Figure 13.**
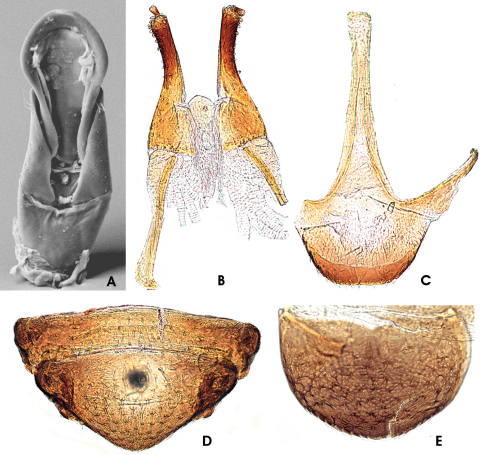
*Ancyronyx pseudopatrolus* Freitag & Jäch, 2007, adult male: **A** aedeagus in ventral few (SEM photograph); adult female: **B** ovipositor in ventral view; **C** sternite VIII in ventral view; **D** ventrite 4 & 5 in ventral view; **E** tergite VIII in dorsal view.

This species was originally described based on a single male exemplar. As additional material including females have been collected since then we will provide a short diagnosis of the female characters and sexual dimorphisms. Furthermore, the aedeagus (Fig. 13A) is figured in here as SEM micrograph, for the detailed diagnosis see Freitag & Jäch (2007), pp. 41 & 46; Figs 12a, b.

#### Adult female diagnosis. 

Ovipositor as in Fig. 13B. Total length c. 500 µm. Stylus comparably stout as in *Ancyronyx punkti* (see Freitag & Jäch, 2007, Fig. 13d), slightly conical towards base (broader apically), slightly outwards directed. Coxite long and slender as in *Ancyronyx patrolus* (comp. Freitag & Jäch, 2007, Fig. 11d); setae rather short, peg-like, apically rounded, not acute, most densely dispersed at coxite apex; mesal coxite margin moderately pubescent; basal portion c. half as long as distal portion, with slightly more acute and pointed setae than those at distal portion, most densely set at proximal and lateral margins. Valvifer about as long as coxite; fibula enlarged and curved inwards at proximal end.

#### Secondary sexual characters.

 Sternite VIII in female (Fig. 13C) overall very similar to that of *Ancyronyx punkti*, with median strut apically widened and truncate; posterior portion with disc densely covered with small, inconspicuous setae; posterior margin with moderately long trichoid setae. Sternite VIII in male weakly sclerotized, median strut distinctly shorter than in female. Ventrite 5 in female (Fig. 13D) overall very similar to that of *Ancyronyx punkti*, subtriangular, with small and only slightly elevated lateral projections. Ventrite 5 in male (see Freitag & Jäch, 2007, Fig. 12d) suboval, stouter and with large and distinctly elevated lateral projections. Tergite VIII in female (Fig. 13E) longer than broad; condyles more or less straight; asperities only conspicuous laterally.

#### Larval diagnosis (based on 6^th^ instar).

Colour (Fig. 5) somewhat similar to that of *Ancyronyx minerva* and *Ancyronyx punkti*, but differs slightly in the dorsal and leg colour patterns. Anterior yellow pronotal band very broad, reaching up to anterior 0.4, sublaterally extended posteriad (area of explanate lateral pronotal gutter). Meso- and metanotum and abdominal segments with paler entire posterior margin or entirely dark brown (except for paler posterolateral projections). Colour patterns of head and abdominal segment IX as in *Ancyronyx punkti*. Legs yellowish pale except for dark distal tibia area around claw insertion.

**Figure 14.**
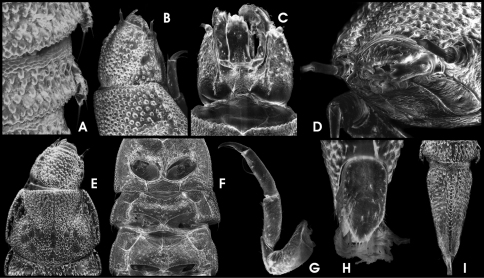
*Ancyronyx pseudopatrolus* Freitag & Jäch, 2007, larva (SEM photographs): **A** lateral detail of abdomen, dorsal, with posterolateral projections and spiracles; **B** anterior portion of pronotum and head, lateral; **C** head, ventral; **D** head, frontal; **E** pronotum and head, dorsal; **F** thoracic segments, ventral; **G** foreleg, ventral; **H** distal portion of abdominal segment IX with operculum, ventral; **I** abdominal segment IX, dorsal.

HW c. 0.29 mm; entire larva about 3.0 mm long. Body shape as in *Ancyronyx punkti*, except for the following characters:

All abdominal posterolateral projections (Fig. 14A) very prominent, distinctly overreaching posterior segment margins. Setiferous tubercles at dorsal site very prominent and protruding (Figs 14A, B). Ventral side densely covered with asperities and scattered setiferous tubercles (Fig. 14E).

Head (Figs 5; 14B–D) broadest at c. posterior 0.3, margins not subparallel. Lateral head with clumped stemmata arranged in well defined round, protruding glabrous area (Fig. 14B); lateral setae short and rather inconspicuous; dorsolateral pair of double setae present and long (Fig. 14B); frontal suture V-shaped (Fig. 5). Labium (Fig. 14C) as in *Ancyronyx punkti*, except for setae and spines of the mentum: sublateral trichoid setae moderately long; lateroapical pair of spines slender, inserted subapically. Antennae, labrum (both Fig. 14D), maxillae and gula (both Fig. 14C) as in *Ancyronyx punkti*.

Pro-, meso- and metathorax (Figs 5; 14E, F) distinctly narrower anteriorly. Pronotum with conspicuous small signa in posterior half arranged as in Fig. 14E.

Sublateroposterior portions and anteriomedian sclerites of thoracic venters with setiferous tubercles (Fig. 14F); remaining ventral areas densely covered with asperities.

Legs (Fig. 14G) as those of *Ancyronyx punkti*.

Abdomen (Figs 5; 14A, H–I) with dorsosagittal carinae at the posterior portions of segments I–VIII and most distinct at the almost entire segment IX (Fig. 14I). Squamose setae at posterior rim of segments I–VIII broken off in specimen figured under SEM, but generally developed (Fig. 5). Sagittal ridge of ventral sclerite of segment I longer than 1/2 of segment length. Apex of segment IX slightly emarginate. Operculum (Fig. 14H) medially deeply impressed, rugulose, with few setae at disk.

#### Variation between larval instars.

 For this species, quite a number of prefinal instar specimens is available. They vary from the final instar description by the overall paler colour, relatively longer posterolateral projections, relatively shorter and broader legs and fewer setae on tibiae and femora.

#### Larval differential diagnosis.

The species can most easily be distinguished from its congeners *Ancyronyx minerva* and *Ancyronyx punkti* by the colour pattern of the anterior pronotum and the dorsal posterior margins of thoracic and abdominal segments and the more distinctly developed, protruding tubercles and of the dorsal crest of the abdominal segment IX.

#### Distribution. 

Only known from few rivers in central Palawan.

### Ancyronyx patrolus

Freitag & Jäch, 2007

http://species-id.net/wiki/Ancyronyx_patrolus

Figs 6 15A–K

Ancyronyx patrolus  Freitag & Jäch, 2007: 41, 44–46 (adult description).

#### Material examined.

 1♂ (ZSM [FR032]) PHIL.: Busuanga, Coron, San Nicolas/Borac, “7Falls" mount. creek; riffle & pool; gravel, boulders, CPOM, sec. forest/rural; c.50m asl, 12°03'11"N, 120°15'28"E 02.2.1995, leg. Freitag (165)M; 4L (0.23, 0.31, 2 × 0.32) (CFP) “PHIL.:Palawan, El Nido, Bgy.Pasadeña Nagkalit-Kalit Falls, small mountain river, degr. prim. forest, rocks, gravel, wood litter, c. 11°15'N, 119°26'E 14.10.1994, leg. Freitag (112)M"; 1L (0.19) (CFP) “PHIL.: Palawan, P. Princesa Panaguman R. 10°15'09"N, 118°58'03"E 17.5.2001, leg. Freitag (PR1)D“; 21 exs.; 1L (0.30) (NMW) “PHIL.: Palawan, P.Princesa; Concepcion, Taranaban R.; c.6km upstr. Highw., mount.riv., riffle; boulders, woodlitter; c.150m asl 10°02'30"N, 119°00'45"E, 20.1.1995 leg. Freitag (16b)M“; 2L (0.30, 0.32) (CFP) “PHIL.: Palawan, P.Princesa; Concepcion, Taranaban R. trib.;mount.creek c.8km upstr.; dist. prim. forest; riffle;rocks, boulders,roots; c.450m asl 10°05'N, 119°01'E, 28.1.1995 leg. Freitag (16f)M"; 2♀♀, 1L (0.26) (CFP, SMTD) “PHIL.: Palawan, P.Princesa; Concepcion, Tagpaya, Camp Aga, Taranaban R. trib.; mount.creek c.8km upstr. Highw., dist. prim. forest; riffle;rocks, boulders, roots; c.450m asl 10°05'N, 119°01'E, 26.4.1995 leg. Pangantihon (16f)M“; 1L (0.30) (CFP) “PHIL.: Palawan, P. Princesa, Bacungan, Bisor Riv.,6km NW Highw. km23, mount. riv., sec. forest; riffle, boulders, gravel, wood, mos; algae, c.9°57'42"N, 118°41'47"E 22.1.1995, leg. Freitag (140b)M";15L (0.26, 0.27, 2 × 0.30, 5 × 0.31, 0.32, 5 × 0.33) (NMW) “PHIL.: Palawan, P. Princesa, Irawan River, 6km NW of PPC, 2km upstream of water plant 9°49'N, 118°39'E 06.4.1994, leg. Freitag (60)M"; 1L (0.25) (CFP) “PHIL.: Palawan, P. Princesa, Iwahig, Salomon Riv.; rural near sec. forest, riffle, boulders, gravel, wood; 9°46'59"N, 118°40'53"E 24.1.1994, leg. Freitag (159)M“; 1♂, 4♀♀, 18L (0.20, 0.29, 0.21, 0.30, 6 × 0.32, 6 × 0.31, 2 × 0.33) (NMW, ZSM [FR005, FR006]) “PHIL.: Palawan, P. Princesa, Iwahig, Balsahan Riv., upstr.dam; riffle, boulders, gravel, wood, moss; 9°46'36"N, 118°39'55"E 24.1.1995, leg. Freitag (20)M"; 5L (0.25, 0.28, 2 × 0.30, 0.31) (PCSD) “PHIL.: Palawan, P.Princesa; Junction to Napsan, 8km SW PPC; 20m S Binuan Bridge,gravel, root packs, riffle, sec.veget. c.20m asl 9°43'N, 118°40'E, 08.2008 leg. Freitag (22b)M“; 3L (0.26, 0.30, 0.32) (SMTD, ZSM) “PHIL.: Palawan, Aborlan; Cabigaan, Talakaigan R.; mount.Riv. upst.dam, riffle, rocks, boulders,CPOM,forest, c.50m asl, 9°26'50"N, 118°26'49"E 25.2.1995, leg. Freitag (154)M"; 1♀, 4L (0.28, 2 × 0.31, 0.32) (SMTD) “PHIL.: Palawan, Narra, 7 km N town centre, downstr. Estrella Falls, mountain riv.; sec. forest, gravel, boulders, submerged wood, riffle; c. 50m asl., c. 9°20'N, 118°23'E 16.4.2010, leg. Freitag & Pangantihon (180a)“; 1L (0.30) (CFP) “PHIL.: Palawan, Narra, 5 km W town proper, Taritien River, riffle, boulders, gravel, leaf litter c. 100m asl. 9°19'11"N, 118°22'35"E 17.4.2010, leg. Freitag et al. (182a)“.

#### Larval diagnosis (based on 6^th^ instar). 

Colour as in Fig. 6: almost entirely dark-brown; antennae, area of explanate lateral thoracic and abdominal gutter, lateral head portions and legs (except for darkened area around claw insertation) pale brown or yellowish. Mouthparts and surrounding portions of head capsule almost black. Without pale pattern at dorsal thoracic and abdominal segments; only apex of abdominal segment sightly paler. Ventral side pale brown.

**Figure 15.**
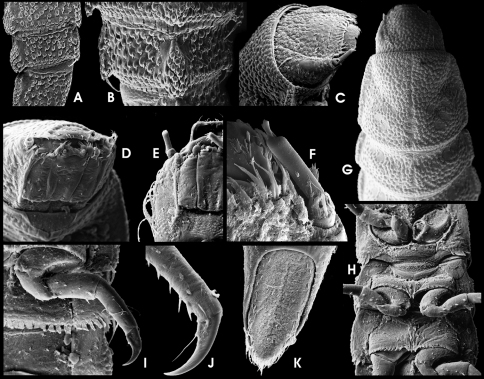
*Ancyronyx patrolus* Freitag & Jäch, 2007, larva (SEM photographs): **A** abdominal segments VII–IX, lateral, with dorsosagittal carinae (left), **B** abdominal segment VIII, dorsal, with drop-shaped protuberance of dorsosagittal carina, posterolateral trichoid tooth and spiracle, **C** head, dorsolateral, **D** head, frontoventral, **E** head, ventral, **F** antenna and lateral portions of clypeus with subbasal fringe of fasciculate setae, dorsal, **G** head, pronotum and mesonotum with signa, dorsal, **H** thoracic segments, ventral, **I** hindleg and first abdominal segments, ventral, **J** tibia and claw of last instar foreleg, lateral, **K** operculum, ventral.

HW 0.32 mm; entire larva up to 3.5 mm long. Body shape as in *Ancyronyx minerva*, except for the following: dorsosagittal carinae at the posterior portions of abdominal segments IV–VIII and the entire length of segment IX very distinct (Figs 15A, B). Setiferous tubercles at dorsal side very prominent and protruding (Figs 15A, B) as in *Ancyronyx pseudopatrolus*. Ventral side smoother, with scattered setae (Fig. 15I).

Head as in Fig. 6 and Figs 15C–F, similar to that of *Ancyronyx pseudopatrolus*. Glabrous area with stemmata slightly exposed. With few short, acuminate setae on each side and a dorsolateral pair of long double setae (Fig. 15C). Frontal suture U-shaped (Fig. 6). Fasciculate setae at subbasal fringe of clypeus very long (Figs 15C, F). Gula (Fig. 15D), labium (Figs 15D, E), maxillae (Figs 15D, E) and labrum (Figs 15D, F) as in *Ancyronyx minerva*. Antennae (Fig. 15F) as in *Ancyronyx punkti*.

Pro-, meso- and metathorax as in Figs 6, 15G and Fig. 15H. Dorsal thoracic segments with conspicuous small round signa (arranged as in Fig. 15G) and a distinct sagittal line (sagittal line partly fused with signa). Thoracic venters (Fig. 15H) rather smooth as in *Ancyronyx minerva*. Legs (Figs 15H–J) similar to those of *Ancyronyx minerva*; inner side of tibiae and femora with longitudinal rim of spinous setae (Fig. 15J); all other parts with scattered squamose setae.

Abdomen (Figs 6; 15A, B, I, K) with distinct dorsal sagittal line and slightly elevated surrounding portions in segments I–IV. Dorsosagittal carinae at the posterior portions of abdominal segments IV–VIII distinctly elevated, somewhat drop-shaped and densely covered with setiferous tubercles (Fig. 15B). Sagittal ridge of ventral sclerite of first segment distinctly shorter than 1/2 of segment length (Fig. 15I). Apex of segment IX emarginate. Operculum (Fig. 15K) elongately subtrapezoidal, medially moderately impressed, rugulose.

#### Variation between larval instars.

 Specimens of 4^th^ and 5^th^ instar vary only little from the final larval instar. The species typical dorsosagittal carinae of the posterior abdominal segments are very distinct. However, the emargination of the abdominal segment IX` apex is not conspicuous, the overall colour is paler, the thoracic and abdominal segments are relatively narrower, the legs are slightly shorter and broader and the setae on tibiae and femora are fewer and not well arranged in rims.

#### Larval differential diagnosis. 

The species can clearly be distinguished from other Palawan species by the lack of pale dorsal colour patches and its highly elevated dorsosagittal carinae which is most conspicuous at the posterior portion of abdominal segment VIII where it appears as a drop-shaped protuberance.

#### Distribution.

Only known from Palawan and Busuanga.

### Ancyronyx montanus

Freitag sp. n.

urn:lsid:zoobank.org:act:A0935C7F-E1D7-4211-8B4F-CDE0A020869A

http://species-id.net/wiki/Ancyronyx_montanus

Figs 7, 8 16A–P 17A–K 

#### Type material. 

**Holotype** ♂ (NMW [FR012]) “PHIL.: Palawan, P.Princesa; Concepcion, Taranaban R. trib.;mount.creek c.8km upstr.; dist. prim. forest; riffle;rocks, boulders,roots; c.450m asl 10°05'N, 119°01'E, 28.1.1995 leg. Freitag (16f)M", terminal parts of abdomen incl. aedeagus glued separately, two tarsi lacking. **Paratypes:** 1♀ (NMW), 1L (0.37) (ZSM [FR038]): same data as holotype; 2♂, 4L (0.30, 0.31, 0.36, 0.37) (SMTD, UPLB, NMW, CFP) “PHIL.: Palawan, Roxas, Bgy.Dumarao downstr. New Rizal Falls, sec. forest, c. 120m asl; boulders, sand, CPOM; 10°28'10"N, 119°19'52"E 05.12.1994, leg. Freitag (135)M".

#### Adult description. 

Body 1.82–1.88 mm long (CL), 0.79–0.83 mm broad (EW), 2.2–2.4 times as broad as wide (CL/EW). Body form elongate, moderately convex dorsally.

Colouration (Fig. 7) predominantly dark brown to black; legs pale brown to dark brown, (articulations slightly darker); claws and antennae pale brown (distal antennal segment darker); elytra without pale patches; ventral side slightly paler than dorsal, but still dark brown.

**Figure 16.**
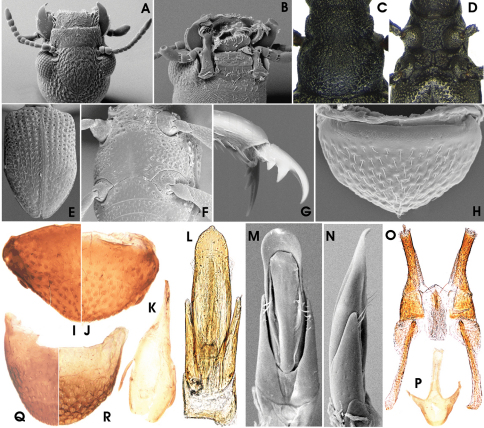
*Ancyronyx montanus* Freitag & Balke, sp. n., (SEM photographs with grey background, stereo microscope photographs with white background) adult male: **A** head, dorsal, **B** head, ventral, **C** pronotum, dorsal, **D** pro-, meso-, and metaventrite, ventral, **E** elytra, dorsal, **F** meso- and metacoxae, metaventrite, ventrites 1–2, ventral, **G** hind claw, **H** ventrite 5, ventral; adult female: **I** ventrite 5, ventral; adult male: **J** ventrite 5, ventral, **K** sternite IX, **L** & **M** aedeagus, ventral, **N** aedeagus, lateral; adult female: **O** ovipositor, ventral, **P** sternite VIII, ventral, **Q** tergite VIII, dorsal; adult male: **R** tergite VIII, dorsal.

Head (Figs 7; 16A, B) 0.41–0.45 mm broad (HW); ID 0.28–0.26 mm; labrum (Fig. 16A) micropunctate, moderately densely covered with long and trichoid setae; frons (Fig. 16A) punctate; clypeus (Fig. 16A) reticulate, moderately densely pubescent; frontoclypeal suture almost straight, slightly impressed. Eyes protruding (Figs 16A, B). Antennae (Fig. 16A) 11–segmented, slender, very slightly longer than head broad. Gula (Fig. 16B) microreticulate, moderately pubescent; gular sutures inconspicuous.

Pronotum (Figs 7; 16C) 0.54–0.65 mm long (PL), 0.52–0.57 mm broad (MW), slightly longer than wide (PL/MW), widest at about posterior 0.2, distinctly narrower than elytra, anteriorly attenuate; anterior margin slightly acuate; margin between pronotum and hypomeron inconspicuous; anterior transverse groove distinct and moderately deeply impressed, medially shallower; anteriorly and posteriorly of transverse groove gently vaulted; posterolateral oblique grooves shallow, inconspicuous; pronotal surface entirely distinctly reticulate; hypomeron reticulate. Prosternum (Fig. 16D) reticulate; prosternal process distinctly narrower medially, medially impressed, posterior margin obtuse.

Scutellum subpentagonal, anteriorly slightly impressed, glabrous. Elytra (Figs 7; 16E) elongate, 1.16–1.35 mm long (EL), c. 1.6–1.8 times as long as wide (EL/EW), almost parallel-sided in anterior 0.1–0.7, posteriorly roundly convergent to apices; elytral apices separately rounded; with c. 10 longitudinal, quite regular, deeply impressed rows of punctures (five strial rows between suture and shoulder); punctures large and deeply impressed; interstices and intervals convex, granulous; lateral elytral gutter moderately broad; humeri prominent. Mesoventrite (Fig. 16D) very short, with longitudinal impression, with few deep punctures. Metaventrite (Fig. 16F) prominent, with distinct discrimen, subanteriorly and medially impressed along the groove, lateral parts punctate to reticulate; anepisternum 3 prominent, with two irregular rows of punctures. Hind wings present; venation not examined.

Legs slightly longer than body; pro- and mesocoxae large, globular; metacoxae only slightly protruding laterally; femora, tibiae, and tarsi (except distal tarsal segment) covered with elongate setiferous tubercles; tibiae distally with a distinct rim of setae; claws (Fig. 16G) well developed, rather gently curved; base of each claw with three teeth, distal one largest, basal one shortest.

Ventrites 1–4 almost glabrous, posteromedially punctate, reticulate anteriorly and laterally; ventrite 5 (Figs 16H–J), moderately densely covered with short adpressed setae emerging from flat tubercles; lateral projection small and inconspicuous.

Sternite IX (spiculum gastrale) as in Fig. 16K; apical margin almost straight, only very slightly emarginate; paraprocts short, not reaching apical margin.

Aedeagus (Figs 16L–N) very similar to that of *Ancyronyx punkti* (see Freitag & Jäch, 2007: Figs 13a, b), but distinctly larger, 440–490 µm long. Median lobe moderately long and slender, distinctly widened subapically (c. 80 µm broad), slightly curved ventrad, with numerous distinct microtube-like structures ending sublateroapically; ventral sac weekly sclerotized; fibula well sclerotized, conspicuous in transillumination (Fig. 16L); corona well developed. Phallobase asymmetrical, slightly widened ventrally, with conspicuous, strongly sclerotized margin; basolateral (penile) apophyses rather short. Parameres elongately subtriangular with basal margin strongly emarginate (lateral view, Fig. 16N), reaching about basal 0.63 of aedeagus, not contiguous ventrally.

Ovipositor (Fig. 16O). Total length c. 620 µm. Stylus slender, slightly bent outwards (partly broken off in specimen examined). Coxite long and slender, distal portion distinctly elongate, with several comparably long, lanceolate setae, most densely set at apex; mesal margin densely pubescent; basal portion c. half as long as distal portion, with same type of setae in distinct patterns as in Fig. 16O. Valvifer about as long as coxite; fibula (mesal, longitudinal sclerotisation) genus typical as in Fig. 16O.

Secondary sexual characters. Sternite VIII in female (Fig. 16P) with median strut apically widened, almost truncate, posterior portion slightly pubescent; sternite VIII in male weakly sclerotized, median strut distinctly shorter than in female. Ventrite 5 in female subtriangular (Fig. 16I), in male (Fig. 16J) shorter and suboval. Tergite VIII in female (Fig. 16Q) longer than broad; condyles more or less straight and prominent; reticulations only conspicuous laterally. Tergite VIII in male (Fig. 16R) broader than long, reticulation more developed than in female covering apical half; basal half patterned with lines of asperities; condyles not distinctly curved, overreaching anterior margin.

**Figure 17.**
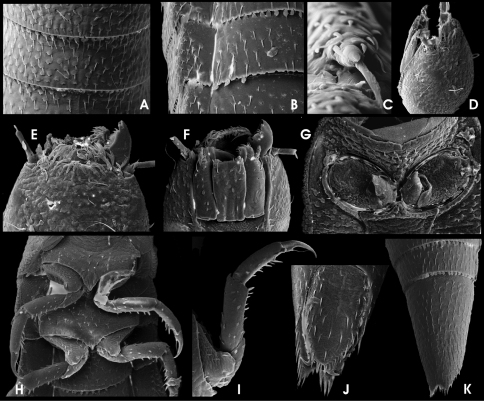
*Ancyronyx montanus* Freitag & Balke, **sp. n.**, larva (SEM photographs), **A** abdominal segments VI–VIII, dorsal, **B** lateral and parts of median sclerites of abdominal venter, with different types of setae and lateral projections, **C** posterolateral abdominal projection with trichoid tooth, **D** head, dorsolateral, **E** head, dorsal, **F** head, ventral, **G** prothorax, ventral, **H** thoracic and first abdominal segments, ventral, **I** hindleg, lateral, **J** operculum, ventral, **K** abdominal segments VIII and IX, dorsal.

#### Adult differential diagnosis.

 *Ancyronyx montanus* superficially resembles dark specimens of *Ancyronyx patrolus* (Freitag & Jäch 2004: Fig. 4), from which it can be easily distinguished by the larger size, slenderer body (EL/EW), the entirely reticulate pronotal surface that lacks any glabrous areas, the medially narrowed prosternal process and its genital characters.

#### Larval description (based on 6^th^ instar).

 Colour as in Fig. 8: very similar to that of *Ancyronyx patrolus*, but overall darker and somewhat shiny; legs entirely pale brown to yellowish. Mouthparts and surrounding portions not distinctly darker than dorsal and ventral head portions. Without pale pattern at dorsal thoracic and abdominal segments; only apex of abdominal segment slightly paler. Ventral side pale brown.

HW 0.37–0.41 mm; entire larva up to 3.3 mm long. Body shape as in *Ancyronyx patrolus*, except for the following: entire body more vaulted in cross section; dorsal sagittal area flat, with rather indistinct sagittal line, without carina at all thoracic and abdominal segments. Setiferous tubercles at dorsal side rather flat, not as elevated as in the other species (Fig. 17A), those of ventral side very flat and inconspicous (Fig. 17B). Setae originated from tubercles (Figs 17A–C) lanceolate to trichoid and distinctly longer than in the previous species. Posterolateral abdominal projections comparably small, attenuate, distinctly larger at anterior abdominal segments (Figs 8, 17C). Trichoid teeth not reaching middle of subsequent abdominal segment when that retracted.

Head (Figs 8, 17D-F) basically as in the previous species, but with the following varying characters: broadest posterior 0.25, laterally slightly convex. Glabrous area with stemmata not exposed. With few long, trichoid setae on lateral sides and one dorsolateral pair of long double setae (Figs 17D–F). Entire head more or less densely covered with squamose to fascicular setae originated from slightly elevated tubercles and additionally with several moderately long trichoid setae (Figs 17E–F). Frontal suture V-shaped. Fasciculate setae at subbasal fringe of clypeus very long, overreaching labrum (Fig. 17E); labrum with large fasciculate setae (Fig. 17E). Gula, maxillae and labium (Fig. 17F) almost as in *Ancyronyx minerva*, but with few squamose setae and shorter maxillary and labial palpi. Mandibles as in Fig. 17E. Antennae (Fig. 17E) almost as in *Ancyronyx patrolus*, scapus with large plumose setae.

Pro-, meso- and metathorax as in Figs 8 and 17G, H. Thoracic terga with small round signa in sublateral portions; sagittal line rather inconspicuous. Thoracic venters (Figs 17G, H) with asperities and irregularly distributed setiferous tubercles; anterior sclerites and posterior portion of lateral sclerites of prothorax (Fig. 17G) densely covered with tubercles. Posteromedial sclerite bald and rugulose. Meso- and metathorax with scattered setiferous tubercles at distal portions of all sclerites, lateral sclerite portions densely covered with asperities (Fig. 17H). Legs (Fig. 17I) similar to those of *Ancyronyx patrolus*, but with comparably long and slender femora (c. as long as tibiae); coxae with distinct asperities; other surfaces rather glabrous. Claws slender and long, moderately bent (Fig. 17I).

Abdomen (Figs 8; 17A–C, J–K) without any elevations or carinae at dorsal sagittal area; segments I–VI with a pair submedian trichoid setae, that are distinctly longer than surrounding lanceolate to trichoid setae (Fig. 17A). Ventral sclerite of first segment without sagittal ridge (Fig. 17H). Apex of segment IX distinctly emarginate (Fig. 17K). Operculum (Fig. 17J) subovate, medially slightly impressed, with scattered lanceolate setae in apical and lateral portions.

#### Variation between larval instars. 

The two specimens of prefinal instar stage vary most conspicuously from the above description by the overall paler brown colour, the more conspicuous dorsal setiferous tubercles that let the pronotal signa appear clearly as well as the slightly crested (in cross-section subtriangular) abdominal segment IX.

#### Larval differential diagnosis.

The species is easily distinguishable from other Palawan species by the lack of any dorsosagittal carinae or elevations, the dark, shiny dorsal colour, its rather large size and the rather shallow dorsal tubercles bearing comparably long, lanceolate to trichoid setae.

#### Distribution.

Only known from the type locality in central Palawan and one site in northern Palawan (Fig. 20).

#### Etymology.

The species is named in reference to the remote mountainous river habitats where it was exclusively recorded from.

### Ancyronyx procerus

Jäch, 1994

http://species-id.net/wiki/Ancyronyx_procerus

Figs 9 18A–J

Ancyronyx procerus  Jäch, 1994: 611–613 (adult description); Jäch, 2003: 259 (new records).

#### Material examined.

 1 L (0.61) (ZSM [FR014]) “PHIL.: Busuanga, Coron; Guadelupe, Balolo R./Brdg. Nat.Rd. km 14; lowld. creek; sec.veget.; run, gravel, CPOM, c.10m asl, 12°01'43"N, 120°06'48"E 03.2.1995, leg. Freitag (169)M"; 1L (0.63) (NMW) “MALAYSIA, Sarawak, Mulu NP, Long Iman 4.3.1993 leg. M. Jäch (20)".

#### Larval description (based on 6^th^ instar).

Colour (Fig. 9) predominantly brown; head distinctly darker to almost black dorsally; lateral head, antennal scape, anterior pronotal collar and legs whitish or yellowish pale. Entire ventral side (except for parts of genae), antennal pedicel, anterior margin of pronotum, claws, lateral abdominal projections, small medioposterior areas or entire posterior margin of thoracic and abdominal segments and a median middle portion of abdominal segment IX pale brown to yellowish.

**Figure 18.**
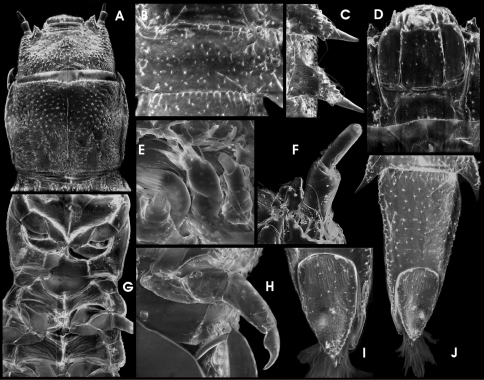
*Ancyronyx procerus* Jäch, 1994, larva (SEM photographs): **A** pronotum and head, dorsal, **B** detail of abdominal segment III, ventral, **C** abdominal posterolateral projections, ventral, **D** head, ventral, **E** apical portions of labium and maxilla, ventral, **F** antenna, ventral, **G** thoracic segments, ventral, **H** hindleg, ventral, **I** operculum, ventral, **J** abdominal segment IX, ventral.

HW c. 0.62 mm, entirely c. 3.7 mm long. Body flattened dorsoventrally, moderately vaulted dorsally, almost flat ventrally, with sagittal line (longitudinal groove from prothorax at least up to 5^th^ abdominal segment). Dorsal side moderately densely covered with setiferous tubercles (Fig. 18A). Ventral side smoother, with scattered setae and few setiferous tubercles (Fig. 18B). Retractable portions of body segments and pronotal collar without setae and tubercles (Fig. 18A). Lateral margins of abdominal segments I–VIII produced laterad forming posterolateral-directed conical projections (Fig. 18C). Projections increasing in size caudad, those of segment VIII c. 3.5 times as long as such on segment I. Rather inconspicuous spiracles present laterally on mesothorax and abdominal segments I–VIII.

Head (Figs 18A, D–F) subquadrate, partly retractable, distinctly prognathous, with three anterior-dorsad directed, pointed projections, one each side between antenna and clypeus and one at median frons (Fig. 18A). Frons rather glabrous, only with small and scattered setiferous tubercles. Stemmata arranged as single lateral spot in a glabrous area, slightly exposed. One irregular rim of moderately long setae at ventrolateral head margin (not visible in dorsal view). Frontal suture broadly V-shaped. Frontoclypeal suture uneven, but somewhat straight. Clypeus distally microreticulate, with protuberant anterior seam; without subbasal fringe of setae or tubercles. Ventral side (Fig. 18D) with few scattered setae and an obvious longitudinal crest each side lateral of gula and maxillae. Genae rugose, with asperities and scattered tubercles. Gula with rather inconspicuous asperities. Maxilla (Figs 18D, E) moderately broad; cardo stout, undivided; lateral portion with one median acuminate seta; stipes subrectangular, glabrous, with few short and one long latero-subapical trichoid setae; maxillary palpus (Fig. 18E) four-segmented, approx. as long as stipes broad, distal segment smallest, cylindrical with several apical sensilla of various shape; predistal segment with lateroapical trichoid seta; galea and lacinia subequal in length and shape, approx. as long as palpus, apically with sensilla. Labium (Figs 18D, E) with broad (about 1.7 times of stipes) mentum (postmentum), broadest at basal half, with median groove most depressed posteriorly, with one pair of moderately long trichoid setae sublaterally at anterior 0.25, one subbasal pair each of spinose and trichoid setae and one pair of short apical lateral teeth. Submentum (prementum) short, transverse, apically convex, with sagittal ridge and one laterobasal pair of setae; ligula inconspicuous with various setae and pegs; labial palpi short, with short and stout palpifer; apical segment similar to that of maxillary palpi, preapical segment with lateral tuft (Fig. 18E). Mandibles not examined. Labrum c. 3 times as wide as long, anterior margin distinctly convex, with a subapical fringe of ramose setae and scattered trichoid and truncate (sensory) setae, proximal portion glabrous. Antenna (Fig. 18F) three-segmented, c. 1/2 as long as head. Peduncle short, about as long as broad, without (visible) dorsolateral fringe of branched trichoid setae; scape cylindrical, longer than pedicel and c. twice as long as broad, with few apical trichoid setae; pedicel cylindrically elongate, comparably short, only slightly longer than scape; flagellum and sensorium (broken off in figured specimen) subequal in length, slender, cylindrically elongate, c. five times as long as broad; apex of flagellum with inconspicuous elongate sensillum.

Prothorax subquadrate, almost as long as broad, with round signa (glabrous areas) in posterior half and near depressed sagittal line. Meso- and metathorax subtrapezoidal, distinctly broader than long, distinctly shorter than prothorax (Fig. 9); medial longitudinal groove and lateral rims distinctly produced posterolaterad. Venter of prothorax (Fig. 18G) with five sclerites: two oblique anterior, two lateral, and one posteromedial sclerite. Anterior sclerites subtriangular; procoxal cavity closed posteriorly; lateral sclerites posteriorly extend mesad, anteriorly appearing divided by an oblique incomplete suture. Entire anterior and lateral prothoracic venter glabrous, few setiferous tubercles in posterior portions. Venter of meso- and metathorax (Fig. 18G) with six sclerites: two oblique anterior, two subquadrate sclerites anterolateral, two elongate meso-posterolateral sclerites; coxal cavities open posteriad; setiferous tubercles sparse on lateral portion; medial portions almost glabrous. Membrane connecting pro- and mesothoracic venter largely extended medially, appearing almost as a separate sclerite (like a prosternal process in adults). Posterior margin of anterior sclerites with fringe of setiferous tubercles.

Legs (Figs 18G, H) stout (compared to larvae of the previous species and adults), similar in shape and length, with scattered trichoid sensilla mainly at femora and tibiae. Coxae large, subtrapezoidal; trochanter shorter, elongately subtrapezoidal, rather slender; femora subtrapezoidal, short; tibiae subcylindrical, broadest basal, distinctly narrower than femur, longer than other segments. Claws elongate, moderately bent, with one subbasal presumably trichoid tooth (broken or invisible in specimen examined).

Abdomen (Figs 9; 18B, I, J). Segments I–VIII similar in shape, broadly subrectangular in dorsal view; terga with depressed sagittal line at least from 1^st^ up to 5^th^ segment. Retractable anterior portion with squamose asperities; posterior terga margins with a rim of squamose setae. Remaining median portions of terga more or less equally covered with setiferous tubercles. Ventral sclerites of segments I–VIII subrectangular, extendingly fused with pleural sclerites from 1^st^ to 8^th^ segment; posterior venter margins with a rim of squamous setae. Segment IX (Figs 18I, J) elongate, subconical (broadest subbasally), subtriangular in cross-section; apex broadly rounded, not emarginate; dorsal and lateral portions densely covered with setiferous tubercles; ventral side with scattered short trichoid setae and some long filiform setae sublaterally (most broken off in specimen figured in Fig. 18J). Operculum (Fig. 18I) subtrapezoidal to subtriangular, less than double as long as broad, medially depressed, rugose. Basal half with small longitudinal ridges and scattered sensilla; apical half with squamose asperities and a lateral rim of trichoid setae; the internally inserted pair of hooks rather small. Gill chamber with long, ramose gill tufts overreaching the opercular margin.

#### Variation between larval instars.

 The two final instar specimens available do not allow to draw conclusions about variations between the instars.

#### Larval differential diagnosis.

The species resembles the previous ones only in very general characters, such as the presence of posterolateral appendages and the distribution of spiracles. This larva is, however, not torpedo-like elongate and subsemicircular in cross section, but dorsoventrally somewhat depressed, only slightly vaulted dorsally. By this it rather resembles the species *Ancyronyx variegatus* from North-America than those of the *Ancyronyx patrolus* group (Freitag & Jäch, 2007, p. 58).

#### Distribution. 

Known from Busuanga, Philippines (recent study; Fig. 20), Malaysia, Brunei and Vietnam (Jäch 1994)

### Ancyronyx helgeschneideri

Freitag & Jäch, 2007

http://species-id.net/wiki/Ancyronyx_helgeschneideri

Figs 10 19A–L

Ancyronyx helgeschneideri  Freitag & Jäch, 2007: 55–58 (adult description).

#### Material examined.

 21 exs., 1L (0.45) (PCSD, SMTD, ZMUC, IMRL, CFP, NMW, ZSM [FR007, FR061]) “PHIL.: Palawan, P. Princesa S Manturon, Cabayugan R. 10°09'28"N, 118°53'26"E 05.3.2001, leg. Freitag (CR4)M"; 5L (0.39, 0.45, 0.48, 0.49, 0.50) (NMW) “PHIL.: Palawan, P. Princesa S Manturon, Cabayugan R. 10°09'16"N, 118°52'30"E 21.4.2001, leg. Freitag (CR3)C"; 4L (0.32, 0.37, 0.40, 0.43) (CFP) “PHIL.: Palawan, P. Princesa S Manturon, Cabayugan R. 10°09'16"N, 118°52'30"E 18.10.2000, leg. Freitag (CR3)M"; 2L (0.25, 0.48) (CFP) “PHIL.: Palawan, P. Princesa S Manturon, Cabayugan R. 10°09'16"N, 118°52'30"E 13.2.2001, leg. Freitag (CR3)C"; 1L (0.42) (IMRL) “PHIL.: Palawan, P. Princesa S Manturon, Cabayugan R. 10°09'16"N, 118°52'30"E 04.9.2000, leg. Freitag (CR3)C-P"; 3L (0.41, 0.46, 0.48) (ZSM) “PHIL.: Palawan, P. Princesa S Manturon, Cabayugan R. 10°09'16"N, 118°52'30"E 31.7.2001, leg. Freitag (CR3)C"; 2L (2 × 0.47) (PCSD) “PHIL.: Palawan, P. Princesa S Manturon, Cabayugan R. 10°09'28"N, 118°53'26"E 04.9.2000, leg. Freitag (CR4)C-R"; 5L (SMTD) “PHIL.: Palawan, P. Princesa S Manturon, Cabayugan R. 10°09'28"N, 118°53'26"E 16.10.2000, leg. Freitag (CR4)C";1L (0.46) (ZMUC) “PHIL.: Palawan, P. Princesa S Manturon, Cabayugan R. 10°09'28"N, 118°53'26"E 25.5.2001, leg. Freitag (CR4)D"; 1L (0.32) (NMW) “PHIL.: Palawan, P. Princesa SE Manturon, Karst spring 10°09'29"N, 118°53'30"E 18.10.2001, leg.Freitag (LS4)C"; 1L (0.45) (ZMUC) “PHIL.: Palawan, P. Princesa SE Manturon, Karst spring 10°09'29"N, 118°53'30"E 09.12.2001, leg.Freitag (LS4)C"; 2♂♂, 1♀ (SMTD, CFP) “PHIL.: Palawan, Rizal, Campung-ulay, Kalitawan Riv.; HW km 212.2, sec. veget/forest; slightly polluted, submerged wood in run, c. 30m asl. 9°19'11"N, 118°22'35"E 02.7.2010, leg. Freitag (186)"; 1♂, 2♀♀ (NMW, ZSM: [FR013])“PHIL.: Busuanga, Coron; Guadelupe, Balolo R./Brdg. Nat.Rd. km 14; lowld. creek; sec.veget.; run, gravel, CPOM, c.10m asl, 12°01'43"N, 120°06'48"E 03.2.1995, leg. Freitag (169)M".

#### Larval diagnosis (based on 6^th^ instar).

Colour as in Fig. 10; dorsal head, pronotal disc and abdominal segment IX from apical 0.1 to apical 0.4 dark brown; lateral head, antennae, anterior and lateral pronotal margins, legs (except for tip of claw), lateral abdominal, meso- and metathoracic margins including projections as well as median portion of segment IX pale yellowish; anterior pronotal edges with conspicuous pale pattern that is extending mediad to disc; remaining parts of dorsal thorax and abdomen brown with indistinct pale patterns; the latter most conspicuous as yellowish spot at posterosagittal margin of all segments. Ventral side entirely pale, except for pale brown gula, maxillae, labium and ventral parts of genae.

**Figure 19.**
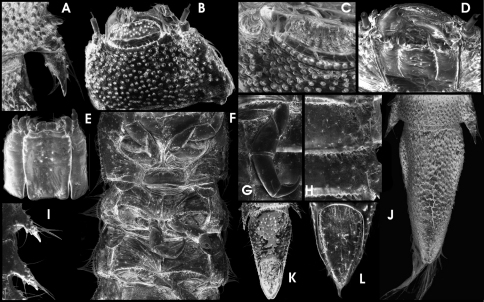
*Ancyronyx helgeschneideri* Freitag & Jäch, 2007, larva (SEM photographs): **A** lateral portion of abdominal segment VI with posterolateral trichoid tooth and spiracle, dorsal, **B** head, dorsal, **C** head portion with labrum, frontodorsal, **D** head, frontoventral, **E** maxillae & labium, ventral, **F** thoracic segments, ventral, **G** hindleg, ventral, **H** portion of abdominal segments, ventral, **I** abdominal posterolateral projections, ventral, **J** abdominal segment VIII & IX, dorsal, **K** abdominal segment IX, ventral, **L** operculum, ventral.

HW c. 0.50 mm, entire larve up to 4.5 mm long. Body shape somewhat similar to that of *Ancyronyx procerus* in the external characters, except for the following characters: spiracles distinctly larger, very prominent (Figs 10, 19A); entire lateral margin with distinct long, trichoid setae (Figs 10, 19F); tubercles at dorsal side more prominent (Figs 19A, B), but dorsal setae very short.

Head (Figs 19B–E) broadest subbasally, slightly conical anteriad, without median pointed projection at frons; pair of sublateral anterior projections between antenna and clypeus rather shallow and inconspicuous (Fig. 19B). Frons moderately densely covered with moderately large and equally dispersed setiferous tubercles; genae rugose, with scattered tubercles; lateral glabrous area with stemmata irregularly shaped. Antenna (Figs 19B–D) short, c. 1/3 as long as head. Scape short, as long as broad, with subapical fringe of stout sensilla; pedicel cylindrical, less than two times as long as scape and c. three times as long as broad, with few inconspicuous apical sensilla; flagellum and sensorium as in *Ancyronyx procerus*. Labrum (Fig. 19C) c. 2.5 times as wide as long; lateroapical edges rounded; dorsal surface with tubercles, ramose setae and short trichoid setae. Ventral head (Figs 19D, E) with well-developed longitudinal crests. Maxilla (Figs 19D, E) almost as in *Ancyronyx procerus*. Labium (Figs 19D, E) with mentum (postmentum) broadest in apical half, one pair of moderately long trichoid setae inserted sublaterally at anterior 0.25; some additional inconspicuous setae present at lateral margin in apical half; pair of apicolateral teeth slender, inserted at a distinct subapical crenation; submentum (prementum) divided.

Prothorax slightly broader than long; tergum with irregularly shaped signa in posterior half. Venter of pro-, meso and metathorax (Fig. 19F) similar to that in *Ancyronyx procerus*, but anterior sclerites more oblique.

Legs as in Figs 19F, G and very similar to those in *Ancyronyx procerus*.

Abdomen (Figs 10; 19A, G–L) with terga slightly depressed groove along sagittal line at least from 1^st^ up to 4^th^ segment; venter almost glabrous, with scattered trichoid setae of different length; segment IX as in Figs 19J–L with emarginate apex; operculum (Fig. 19L) glabrous, with rather inconspicuous basal ridges; disc without conspicuous asperities, covered with few scattered setae.

#### Variation between larval instars.

 Specimens of the 3^rd^ to the 6^th^ (final) instar stage are available for study. Within this range it is obvious that the younger the specimens the paler they are, the longer are all kinds of setae (in relation to the body) and the fewer setiferous asperities are present. Additionally, the limb setae are varyingly arranged and the terminal abdominal segment is relatively shorter and broader as well as the legs. The latter is most obvious between the final and prefinal instar stages.

#### Larval differential diagnosis.

The larvae of *Ancyronyx helgeschneideri* resemble those of *Ancyronyx procerus* but can be clearly distinguished by absence of the pointed projections at median frons, the more shallow projections between the antenna and clypeus, the larger and more protruding spiracles, the more convex head shape, the varying labrum and antennae and the dorsal surfaces of head, thorax and abdomen that are densely covered with larger tubercles as well as the entire lateral body margin bearing long, conspicuous setae.

#### Distribution.

Only known from Palawan and Busuanga.

**Figure 20.**
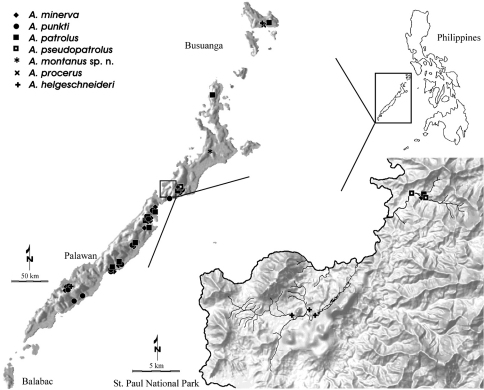
Collecting sites of the *Ancyronyx* species treated herein in Palawan and Busuanga, including enlarged map of St. Paul National Park. See Freitag & Jäch, 2007, Fig. 17 for previous records.

## Key to the larvae of Ancyronyx species of Palawan and Busuanga (without Ancyronyx minutulus)

*Ancyronyx* larvae can be distinguished from other Elmid larvae by the unique combination of the following characters:

Body form subsemicircular in cross-section or dorsoventrally flattened (depressed), with posterolateral projections or processes on abdominal segments I–VIII and lateral spiracles on mesothorax and abdominal segments I–VIII. Ventral prothorax with five sclerites (including one posteromedial sternellum), meso- and metathorax with six sclerites. Abdomen with pleura on segments I–VIII.

**Table:** 

1	Body torpedo-like elongate, subsemicircular in cross section. Posterolateral abdominal projections small, lobate with tip posteriad directed (Figs 3–6, 8)	2
–	Body flattened dorsoventrally (depressed), only slightly vaulted. Posterolateral abdominal projections large, conical with tip posterolaterad directed (Figs 9–10)	6
2	At least terminal segment, but usually several posterior abdominal segments with dorsosagittal carina or elevation (Figs 11K, 12K, 14I). Terminal abdominal segment subtriangular in cross section. Dorsal surface appearing granulate by distinct tubercles (Figs 3–6)	3
–	All abdominal segments without dorsosagittal carinae or elevations (Fig. 17K). Terminal abdominal segment subsemicircular in cross section. Dorsal surface shiny (Fig. 8), tubercles indistinct (Palawan, rare)	*Ancyronyx montanus*
3	Some peripheral areas of dorsal pronotum with obvious pale colour patches (Figs 3–5). Dorsosagittal carinae low, not extended into a drop-shaped protuberance on abdominal segment VIII	4
–	Entire dorsal surface colour without obvious pale patches (Fig. 6). Dorsosagittal carinae high, extended into a drop-shaped protuberance at the posterior portion of abdominal segment VIII (Fig. 15B).(Palawan and Busuanga; common)	*Ancyronyx patrolus*
4	Pronotum anteriorly with small transverse yellow band (less broad than 1/4 of pronotum, Figs 3–4). Posterior third of abdominal segment IX without distinct dorsosagittal carina (Figs 11K, 12K)	5
–	Pronotum anteriorly with broad transverse, rather pale band (extended over c. 1/3 of pronotum, Fig. 5). Entire abdominal segment IX with distinct dorsosagittal carina (Fig. 14K). (Palawan; rare)	*Ancyronyx pseudopatrolus*
5	Anterior yellowish band of pronotum slightly extended posteriad along the midline (Fig. 3). Abdominal apex rounded (Fig. 11K), pale dorsal colour pattern lacking or limited to tip (Fig. 3). (Palawan, Busuanga, Mindoro; common)	*Ancyronyx minerva*
–	Anterior yellowish pronotal band not extended posteriad along the midline (Fig. 4). Abdominal apex truncate or slightly emarginate (Fig. 12K), apical pale dorsal colour pattern conspicuous and extending about one third of terminal segment (Fig. 4). (Palawan, Busuanga, Mindoro; common)	*Ancyronyx punkti*
6	Frons with one median pointed projection (“horn”) (Fig. 18A). (Busuanga and presumably other Philippine islands; presumably rare)	*Ancyronyx procerus*
–	Frons without median pointed projection (Fig. 19B). (Palawan, Busuanga; locally abundant)	*Ancyronyx helgeschneideri*

## Updated check list of the Philippine species of Ancyronyx

1.	*Ancyronyx helgeschneideri* Freitag & Jäch, 2007 (Palawan, Busunga)

2.	*Ancyronyx minerva* Freitag & Jäch, 2007 (Palawan, Mindoro)

3.	*Ancyronyx minutulus* Freitag & Jäch, 2007 (Palawan)

4.	*Ancyronyx montanus* Freitag & Balke, new species (Palawan)

5.	*Ancyronyx patrolus* Freitag & Jäch, 2007 (Palawan, Busuanga)

6.	*Ancyronyx procerus* Jäch, 1994 (Busuanga, Borneo, Vietnam)

7.	*Ancyronyx pseudopatrolus* Freitag & Jäch, 2007 (Palawan)

8.	*Ancyronyx punkti* Freitag & Jäch, 2007 (Palawan)

9.	*Ancyronyx schillhammeri* Jäch, 1994 (Mindoro)

10.	*Ancyronyx sophiemarie* Jäch, 2004 (Sibuyan)

## Discussion

We successfully used mitochondrial DNA sequencing to associate different life stages of beetles with each other, substantiated by morphological description of larvae.Use of DNA sequences has helped to avoid potential pitfalls, as for *Ancyronyx procerus* samples where an unknown larva of a species, which was formerly not recorded in the area, occurred syntopically on the very same piece of wood debris with adults only of another species. This could have led to a misinterpretation as to which larva belongs to which adult.

The morphological species groups suggested by Freitag & Jäch (2007) were supported here by morphological characters of the larvae. Two main types are recognized. The *Ancyronyx patrolus* species group is characterized by smaller size, more vaulted larval body shape (in cross section) and rather small, caudal-directed posterolateral abdominal projections. The larvae of the *Ancyronyx variegatus* species group are larger, depressed (in cross section), and display large, lateral-directed posterolateral projections.

The female and the larva of *Ancyronyx minutulus* Freitag & Jäch, 2007 still remain unknown to science. No additional material has been collected since the single holotype male was described despite intense sampling on Palawan Island including its type locality.

## Supplementary Material

XML Treatment for Ancyronyx minerva

XML Treatment for Ancyronyx punkti

XML Treatment for Ancyronyx pseudopatrolus

XML Treatment for Ancyronyx patrolus

XML Treatment for Ancyronyx montanus

XML Treatment for Ancyronyx procerus

XML Treatment for Ancyronyx helgeschneideri
